# Analysis of the Overpressure Fields in a Shock Tube with Multi-Point Initiation

**DOI:** 10.3390/s23104743

**Published:** 2023-05-14

**Authors:** Zhuo Chen, Huiqi Ren, Qiang Zhao, Songbai Zhou, Zhilin Long, Wei Liu

**Affiliations:** 1 School of Mechanical Engineering and Mechanics, Xiangtan University, Xiangtan 411105, China; 2 Defense Engineering Institute, Academy of Military Sciences, People’s Liberation Army of China, Luoyang 471023, China; 3 College of Civil Engineering, Xiangtan University, Xiangtan 411105, China

**Keywords:** shock tube, overpressure, multi-point initiation, numerical simulation

## Abstract

Shock tubes can carry out dynamic mechanical impact tests on civil engineering structures. The current shock tubes mostly use an explosion with aggregate charge to obtain shock waves. Limited effort has been made to study the overpressure field in shock tubes with multi-point initiation. In this paper, the overpressure fields in a shock tube under the conditions of single-point initiation, multi-point simultaneous initiation, and multi-point delayed initiation have been analyzed by combining experiments and numerical simulations. The numerical results match well with the experimental data, which indicates that the computational model and method used can accurately simulate the blast flow field in a shock tube. For the same charge mass, the peak overpressure at the exit of the shock tube with the multi-point simultaneous initiation is smaller than that with single-point initiation. As the shock waves are focused on the wall, the maximum overpressure on the wall of the explosion chamber near the explosion zone is not reduced. The maximum overpressure on the wall of the explosion chamber can be effectively reduced by a six-point delayed initiation. When the interval time is less than 10 ms, the peak overpressure at the nozzle outlet decreases linearly with the interval of the explosion. When the interval time is greater than 10 ms, the overpressure peak remains unchanged.

## 1. Introduction

Reinforced concrete is widely used in civil engineering structures [1,2]. With the rapid development of the economy, many important economic facilities are threatened by accidental explosions and terrorist attacks [3]. The shock tube is a device that can be used to simulate blast waves, which can test most materials and engineering structures [4,5,6,7,8]. Therefore, investigating the overpressure fields in shock tubes has important engineering significance.

The explosive-driven method is one of the main driving methods used for shock tubes [9]. At present, most shock tubes and similar devices detonate the aggregate charge directly in the explosion chamber to form shock waves. Based on this driving method, scientists have carried out various studies on the design of shock tubes. Stewart designed a conical shock tube that allows the use of smaller charges to obtain stronger shock waves [10]. Sawyer et al. designed an advanced blast simulator that can accurately simulate the shock waves of a free-field explosion and can be used on head models for impact damage testing [11]. Mejia-Alvarez et al. designed a large blast chamber with a logarithmic spiral drive section that can be used to study the impact effects on full-size animal models subjected to blast loading [12]. Ousji et al. performed numerical simulations of the blast flow field of a shock tube and obtained fitted equations for parameters such as explosive mass and device diameter with respect to overpressure and impulse [13]. Li et al. analyzed the effect of the shape and size of each structural component of a shock tube on the shock waveform [14]. Researchers have also carried out various experiments with these shock tubes. Blanc et al. used a shock tube to perform impact tests on sacrificial layers with different granular materials and analyzed the fragmentation process of granular media [15]. Louar et al. used a shock tube to perform impact loading tests on a metallic aluminum plate [16]. They compared the experimental results with those under a free air explosion and analyzed the loading characteristics of shock waves and the dynamic response of aluminum plate. Heshmati et al. studied the impact response of a disc subjected to an underwater blast load using a conical shock tube and analyzed the effect of disc material properties, thickness, and charge mass on the impact deformation [17]. Ren et al. designed a large shock tube [18]. The device has several sub explosion chambers installed in the bottom of the explosion chamber. Multi-point detonation can be achieved by dispersing explosives in each sub explosion chamber. Additionally, they suggested that multi-point detonation could reduce shock wave damage to the shock tube structure. However, no further experimental studies of this shock tube have been reported.

Many studies have been carried out on multi-point detonation in other areas. In a multi-point explosion, the shock waves generated at different locations will meet and superimpose at a certain moment, so that the pressure in the superposition region significantly increases [19]. Lin et al. studied the loading characteristics of shock waves with underwater multi-point initiation and obtained the theoretical equation for shock wave superposition [20,21]. Hu studied the parameters of shock waves and air bubbles in underwater explosions using multi-point arrays initiation [22]. The focus of the shock waves generated by multi-point initiation increased the peak pressure of the shock waves and bubbles. Bai et al. studied the overpressure fields of fuel–air explosive cloud explosions under multi-point initiation. They analyzed the effects of the number of initiation points and the distance between charges on shock wave propagation characteristics and damage performance [23].

Currently, researchers have designed a variety of explosive-driven shock tubes [10,11,12] and have carried out many experimental studies with them [15,16,17]. These studies provide the technical support for the design and application of shock tubes. However, there is little research on the use of multi-point detonation technology for shock tubes. Studies of multi-point detonation in the free field have shown that multi-point detonation causes superposition of shock waves, which increases the intensity and damage range of the shock wave [22,23]. This provides a reference for the application of multi-point initiation technology in the shock tube. In this paper, experiments and numerical simulations have been carried out to analyze the load characteristics of the impact tube under single-point detonation and multi-point detonation. This research provides data to support new shock tube designs.

## 2. Materials and Methods

### 2.1. Computational Model

**Figure 1:**
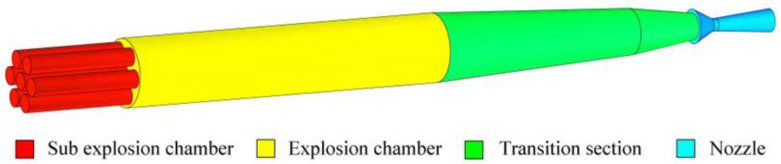
Schematic of computational model used in the shock tube detailing.

Figure 1 is a schematic of the computational model of the shock tube detailing. The shock tube is divided into four parts: sub explosion chambers, explosion chamber, transition section, and nozzle. The explosion chamber is 3 m in diameter and 20 m long. On the left end of the explosion chamber are seven sub explosion chambers. One of the sub explosion chambers is installed centered at the axis of the chamber. The remaining six sub explosion chambers are distributed in a circle at equal intervals. The depth of the sub explosion chambers is 5 m, the external diameter is 1 m, and the internal diameter is 0.8 m. The length of the transition section is 12.53 m. The nozzle length is 3.72 m, and the diameter of the outlet is 1 m.

**Figure 2:**
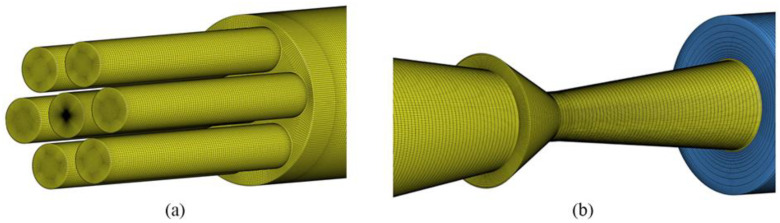
Schematic of computational mesh used in the shock tube simulations. (**a**) Sub explosion chambers. (**b**) Nozzle and outlet area.

As shown in Figure 2a,b, the mesh discretization of the shock tube’s interior and exit areas is carried out. The grid size of the TNT area is 2 mm. The air grid in the shock tube away from the center of the explosion is relatively sparse, with a grid size of 30 mm. The grid size of the air zone outside the shock tube is 60 mm. The initial pressure and initial temperature in the computational domain are set to 101,325 Pa and 303.15 K. The inner wall of the shock tube is defined as a no-slip boundary, and the outlet boundary is defined as a non-reflecting boundary.

As shown in Table 1, six tests and six numerical simulations were performed according to the number of detonation points and detonation interval time. Single-point detonation was used in experiment 1 and simulation 1. In experiment 2, experiment 3, simulation 2, simulation 3, and simulation 4, a multi-point simultaneous detonation was used. Experiment 4, experiment 5, experiment 6, simulation 5, and simulation 6 all used six-point delayed detonation. The cases were computed on a numerical workstation with two Xeon Gold 6152 processors (22 cores, 2.10 GHz). To reduce the computational effort, for the cases of single-point initiation, double-point, and four-point simultaneous initiation, a quarter-symmetric grid is adopted. For the case of five-point simultaneous initiation, a half-symmetric grid is adopted. Finally, for the case of six-point delayed initiation, a full grid is adopted.

### 2.2. Experimental Method

**Figure 3:**
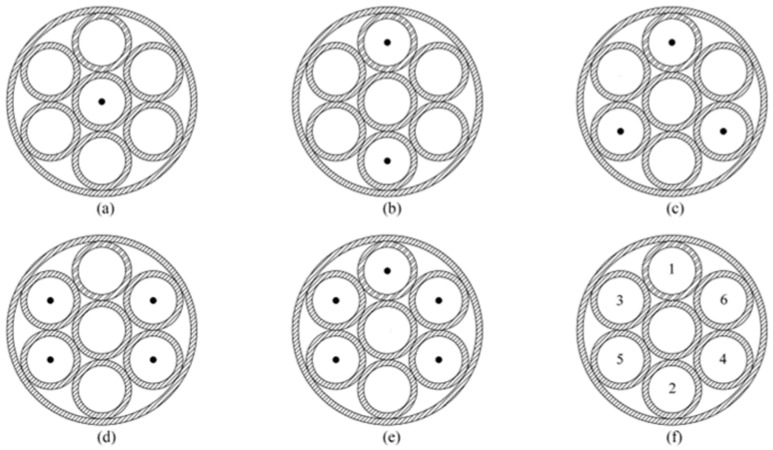
Arrangement of charges. (**a**) Single-point initiation. (**b**) Double-point initiation. (**c**) Three-point initiation. (**d**) Four-point initiation. (**e**) Five-point initiation. (**f**) Six-point initiation.

Figure 3 shows the location of TNT for single-point and multi-point initiation. For single-point detonation, the TNT is in the central sub explosion chamber. For multi-point detonation, the TNT is in the outer sub explosion chambers, and the central sub explosion chamber is empty. The detonation sequence of the TNT charge in the sub explosive chambers when using a six-point delayed detonation is depicted in Figure 3f. Table 1 lists the detonation interval time for delayed detonation. By detonating the TNT in this order, the superimposed effect of the shock waves can be reduced. The charge mass of TNT in all tests and simulations is 1.2 kg.

**Figure 4:**
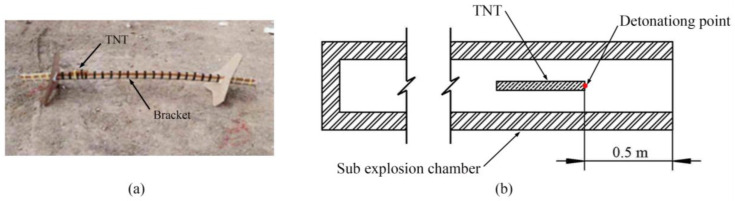
(**a**) TNT charge. (**b**) The location of TNT.

As shown in Figure 4, the TNT is tied to the bracket and placed together in the sub explosion chamber. The TNT is located 0.5 m from the exit of the sub explosive chamber. It is detonated with a detonator. The detonating point is at the right end of the TNT.

**Figure 5:**
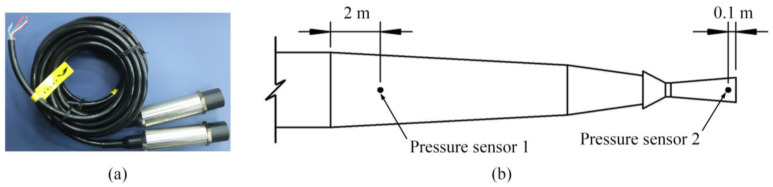
(**a**) Pressure sensors. (**b**) The location of pressure sensors.

As shown in Figure 5, two piezoresistive pressure sensors (CYG401F, Shuangqiao Sensor, Kunshan, China) were installed on the wall of the shock tube. One is in the transition section, which is 2 m from the explosion chamber exit. Another pressure sensor is in the nozzle, which is 0.1 m from the nozzle exit. There is a trigger wire connected between the detonator and measuring computer. The computer starts measuring before the detonation of the explosive. The detonation of the detonator causes the trigger wire to break, thus determining the moment of detonation. The high temperature and pressure gas generated by the explosion of the TNT charge expands and creates shock waves in the explosion chamber. The shock waves can form a relatively uniform wavefront after passing through the transition section, and then reach the exit through the nozzle.

### 2.3. Numerical Method

The Blastfoam solver, based on the open-source software Openfoam, is used for the numerical calculations in this paper [24,25]. The solution is suitable for high detonation, explosion safety, and general compressible flow conditions [26]. It can accurately simulate the explosion flow field in the shock tubes [3]. The numerical simulations use the Euler governing equation based on explosive detonation. The Euler equation consists of the continuity equation, the momentum equation, and the energy equation, which can be expressed by the following formulas:

(1) $$\frac{\partial U}{\partial t}+\nabla \cdot F=S$$

where **U** is the conservation quantity of the volume, mass, momentum, and energy; **F** is the corresponding flux, and **S** is the source term. These terms are defined as the following equations indicate:

(2) $$U=\left(\begin{array}{c} \alpha_{i}\\ \alpha_{i}\rho_{i}\\ \rho u\\ \rho E \end{array}\right),F=\left(\begin{array}{c} \alpha_{i}u_{i}\\ \alpha_{i}\rho_{i}u_{i}\\ \rho u⊗u+pI\\ \left(\rho E+p\right)u \end{array}\right),S=\left(\begin{array}{c} \alpha_{i}\nabla \cdot u_{i}\\ 0\\ 0\\ 0 \end{array}\right)$$

where *α_i_*, *ρ_i_*, and **u***_i_* are the volume fraction, density, and velocity of the *i* phase. *p* is the pressure, *E* is the total energy, and **I** is the unit matrix.

The detonation product of TNT adopts the Jones–Wilkins–Lee equation of state [27]. The expression for the pressure of the detonation product in the JWL equation is given as follows:

(3) $$p=A(1-\frac{\omega }{R_{1}V})e^{-RV}+B(1-\frac{\omega }{R_{2}V})e^{-R_{2}V}+\frac{\omega e}{V},$$

where *p* is the pressure, and *V* is the relative volume. *A*, *B*, *R*_1_, *R*_2_, and *ω* are the empirical parameters of the explosive. *e* is the initial specific energy. Table 2 lists the values of the TNT parameters.

The ideal gas equation of state is used for the air [28]. The expression for the pressure of the air is given as follows:

(4) p=(γ−1)ρe

where *p* is the pressure, *γ* is the ratio of the specific heat, and *e* is the initial specific energy. Table 3 lists the values of the air parameters.

### 2.4. Verification of the Numerical Method

**Figure 6:**
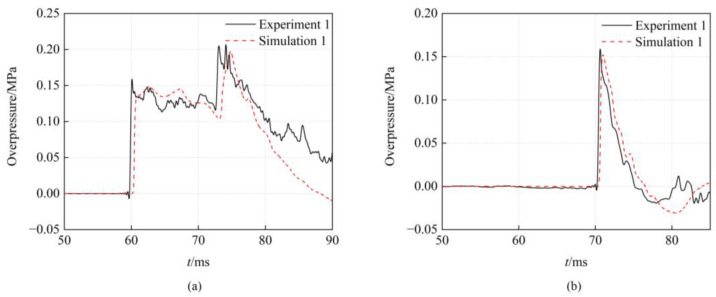
Comparison of the experiment and simulation. (**a**) Gauge 1. (**b**) Gauge 2.

Figure 6 shows the overpressure histories of the simulation and experiment at the gauge 1 and gauge 2. By contrast, the overpressure time curves of the numerical calculation are consistent with the experimental curves. The time–history curve of overpressure in Figure 6a has two peaks. The first peak is the right shock wave from the upstream explosion. The second peak is the reflected shock wave generated at the contraction section of the nozzle when the right shock wave arrives. Table 4 lists the peak overpressure at the gauges for single-point detonation. The difference between the experimental and simulated peak overpressure for both gauges is both 4%. The numerical results are well matched with the experimental results, and the numerical methods can be used to simulate the explosion flow field in the shock tube.

## 3. Results

### 3.1. Shock Waves Propagation Characteristics in Simulations

**Figure 7:**
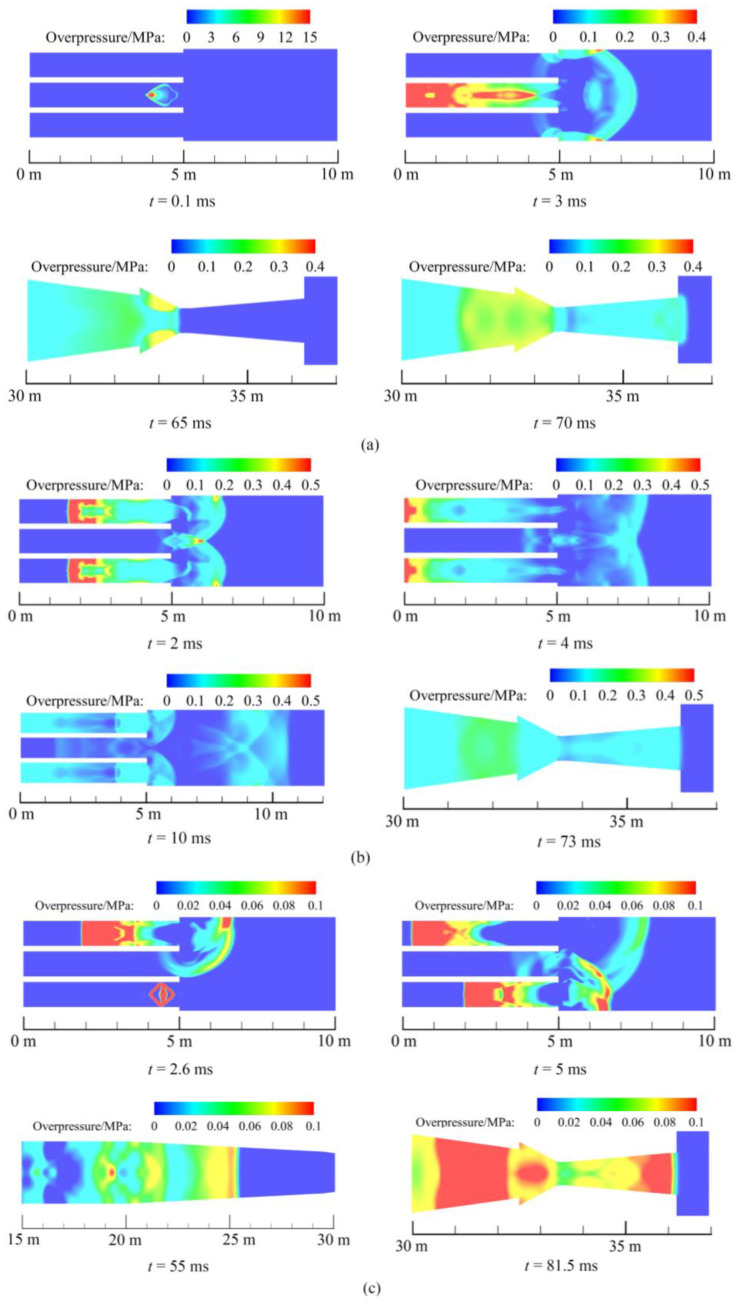
Time evolutions of simulations. (**a**) Single-point initiation. (**b**) Double-point initiation. (**c**) Six-point delayed initiation with 2.5 ms interval.

Figure 7 shows the overpressure cross-section contours of the shock tube with single-point detonation and multi-point detonation. The time of the first detonation is defined as the initial moment. After the explosion of the aggregate charge, a left explosion wave and a right explosion wave were formed in the central sub explosion chamber (Figure 7a). At 3 ms, the right shock wave reflected on the wall of the explosion chamber, and the pressure on the wall was rising rapidly. Due to the diameter of the nozzle inlet being greater than the diameter of the transition section, the shock wave was reflected on the wall of the nozzle contraction section at 65 ms and formed two shock waves to the upstream and downstream, respectively. In the end, the right shock wave reached the pressure sensor near the nozzle outlet at the time of 70 ms.

When a double-point simultaneous detonation was used, shock waves were reflected on the wall of the explosion chamber at 2 ms (Figure 7b). At the same time, 1 m from the exit of the sub explosion chamber at the location of the axis formed a shock wave superposition region. The peak overpressure at this area was 0.55 MPa. The intensity of the shock waves decreased as they propagated. At 10 ms, the maximum overpressure on the axis of the explosion chamber was 0.12 MPa, the maximum overpressure on the wall was 0.19 MPa. This shows that the superposition of the shock waves on the axis does not affect the wall. At 73 ms, the right shock wave eventually reached the pressure sensor near the nozzle outlet.

For the six-point delayed initiation, at the interval time of 2.5 ms, each sub explosion chamber in turn detonated the TNT charges (Figure 7c). Shock waves were focused at 5 ms. Maximum overpressure in the superimposed area was 0.1 MPa. At 55 ms, the first shock wave entered the transition section, followed by several shock waves. This indicates that the delayed detonation results in the shock waves generated by the six sub explosion chambers not all converging into a strong shock wave. At 81.5 ms, the right shock wave reached the pressure sensor near the nozzle exit.

By comparing the results of these three cases, the use of multi-point initiation and delayed detonation weakened the strength of the shock wave. Therefore, the fastest shock wave reached the exit of the nozzle with a single-point initiation, while multi-point simultaneous initiation is the second fastest, and multi-point delayed initiation is the slowest. In addition, the delayed initiation decreased the maximum overpressure in the shock wave superposition region.

### 3.2. Overpressure on the Wall of Explosion Chamber with Simultaneous Initiation in Simulations

**Figure 8:**
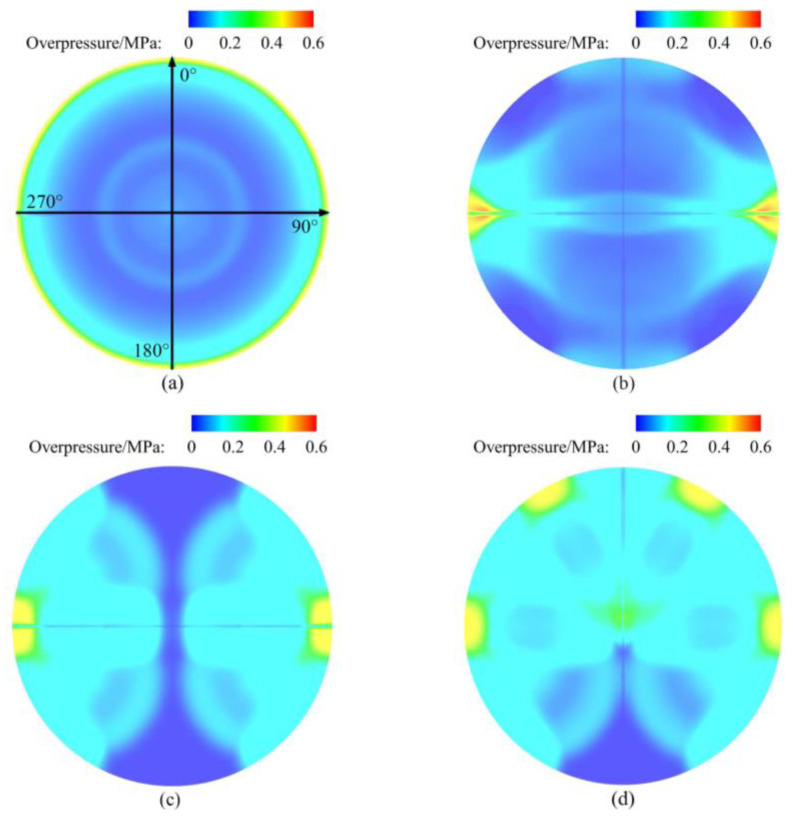
Overpressure contours for simultaneous initiation in simulations. (**a**) Single-point initiation. (**b**) Double-point initiation. (**c**) Four-point initiation. (**d**) Five-point initiation.

Figure 8 shows the overpressure contours of the explosion chamber for single-point initiation and multi-point simultaneous initiation. The profile is 1.5 m from the left end of the explosion chamber. As shown in Figure 8a, the axis of the explosion chamber is the origin, and the 0-degree angle is above it. The angle on the wall of the explosion chamber is determined with a clockwise rotation.

For the single-point initiation, the TNT charge was in the central sub explosion chamber, so that the overpressure distribution on the wall was uniform. The maximum overpressure on the wall was 0.43 MPa. For the multi-point detonation, the TNT charges were located in the outer sub explosion chamber. The shock waves generated by the detonation would propagate along the wall after reflection from the wall, and then meet and converge at a certain position. For two-point detonation and four-point detonation, the shock waves were focused at 90 degrees and 270 degrees. The maximum overpressure in the superimposed area was 0.63 MPa and 0.43 MPa, respectively. In the case of a five-point detonation, the shock waves were focused on the wall at 30, 90, 270, and 330 degrees. The maximum overpressure in the superimposed area was 0.41 MPa.

Multi-point simultaneous detonation can disperse the charge to reduce the intensity of the shock wave generated by a single sub explosive chamber. However, the focus of the shock wave on the wall leads to an increase in overpressure. In the case of two-point detonation and five-point detonation, the maximum overpressure on the wall was 1.5 and 0.95 times that of single-point detonation. Therefore, multi-point simultaneous detonation is not effective in reducing the impact load on the walls of the explosion chamber.

### 3.3. Overpressure on the Wall of Explosion Chamber with Delayed Initiation in Simulations

**Figure 9:**
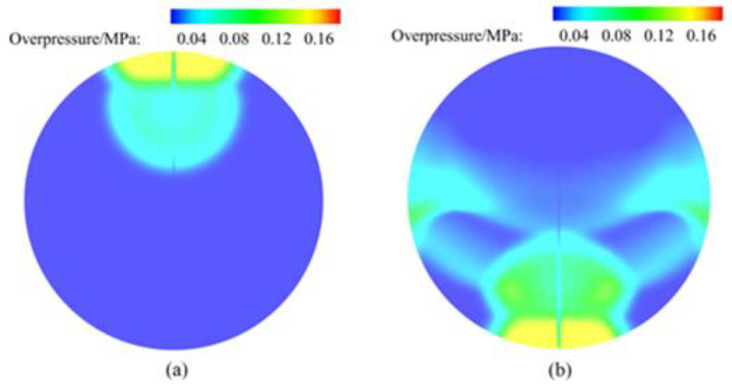
Overpressure contours of simulation 5 (Six-point delayed initiation with 2.5 ms interval). (**a**) 2.6 ms. (**b**) 5 ms.

Figure 9 shows the overpressure contours of the explosion chamber for six-point initiation with 2.5 ms interval. The distance between the sub explosion chambers detonated is long, and the intensity of the shock wave will gradually decrease as it propagates. Therefore, the overpressure in the superimposed region is low. At 2.6 ms, the shock wave of the first detonation was reflected at the wall and the maximum overpressure was 0.15 MPa. At 5 ms, the shock wave of the second detonation was reflected at the wall and the maximum overpressure was 0.16 MPa. The maximum overpressure was 0.37 times that of a single-point detonation. At the same time, the first shock wave met the second shock wave at 96-degree and 264-degree positions on the wall. The maximum overpressure in the superimposed area was 0.09 MPa. If the explosion interval is greater than 2.5 ms and the charge mass of TNT is constant, then it will have no effect on the intensity of the shock waves. Therefore, it will not change the maximum pressure on the wall. In summary, the shock load on the explosion chamber walls can be effectively reduced by multi-point delayed detonation.

### 3.4. Overpressure at the Nozzle Outlet under Multi-Point Simultaneous Initiation

**Figure 10:**
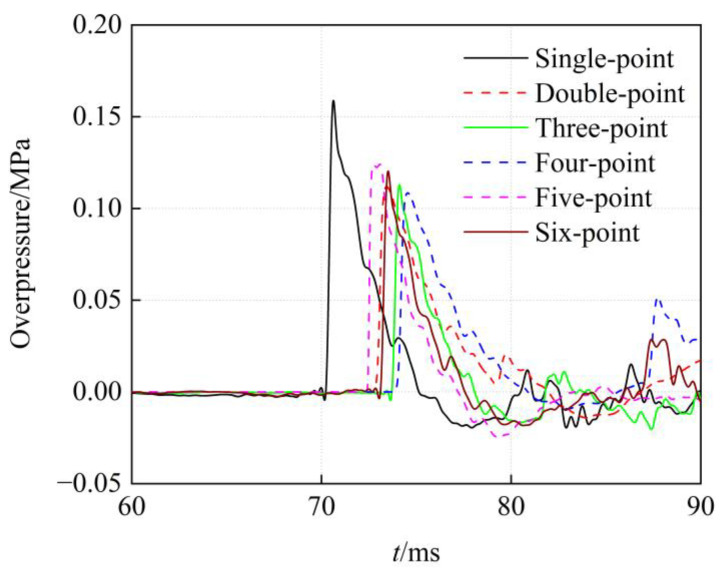
Overpressure history under the simultaneous initiation.

The overpressure history at the nozzle outlet for the single-point initiation and multi-point simultaneous initiation is shown in Figure 10. The curves obtained from the tests are solid lines, and the curves obtained from the simulations are dashed lines. The peak overpressure at the nozzle outlet under single-point detonation is 0.159 MPa. In multi-point simultaneous detonation, the overpressure peak of the four-point detonation is the lowest, and the overpressure peak of the five-point detonation is the highest. The peak overpressure of the four-point detonation is 0.108 MPa, which is 0.68 times that of the single-point detonation. The peak overpressure of the five-point detonation is 0.124 MPa, which is 0.78 times that of the single-point detonation. Multi-point simultaneous detonation disperses the TNT charge, which weakens the intensity of the shock waves and reduces the peak overpressure at the exit of the nozzle. In addition, changing the number of detonation points has less effect on the peak overpressure at the nozzle outlet.

### 3.5. Overpressure at the Nozzle Outlet under Six-Point Delayed Initiation

**Figure 11:**
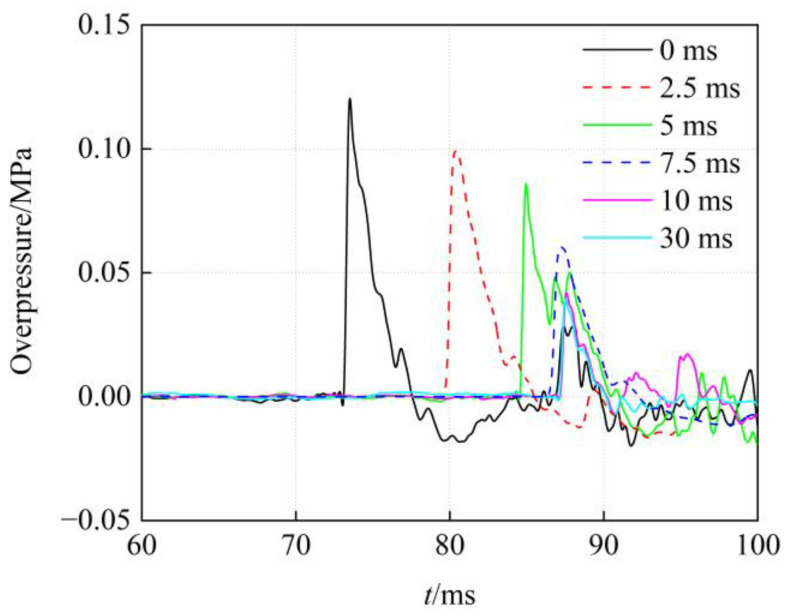
Overpressure time–history under the six-point delayed initiation with various initiation intervals.

Figure 11 presents the overpressure time–history at the nozzle outlet for the six-point delayed initiation with various initiation intervals. The initiation intervals for the six cases are 0 ms, 2.5 ms, 5 ms, 7.5 ms, 10 ms, and 30 ms, respectively. The curves obtained from the tests are solid lines and the curves obtained from the simulations are dashed lines. With 0 ms initiation interval, the overpressure is highest at the outlet. As the detonation interval increases, the peak overpressure at the nozzle outlet decreases linearly. This indicates that due to increasing the detonation time, the shock waves and reflected shock waves generated from sub explosion chambers cannot all converge. When the initiation interval is greater than 10 ms, the overpressure peak value changes very little. This means that the shock wave generated by the second detonation charge cannot catch up with the shock wave generated by the detonation at 0 ms.

**Figure 12:**
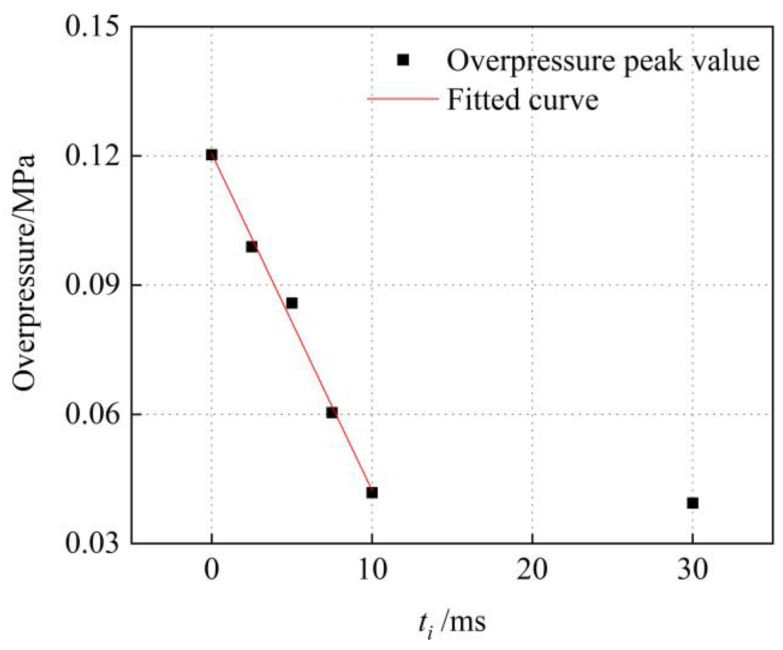
Fitted curve of overpressure on interval time.

Figure 12 demonstrates the fitted curve of the peak overpressure at the nozzle exit under the six-point delayed detonation on the detonation interval. The corresponding formula is as follows:

(5) $$\Delta p=-0.0078t_{i}+0.1205\;0\leq t_{i}\leq 10$$

(6) $$\Delta p\approx 0.0418\;t_{i}>10$$

where Δ*p* is the peak overpressure at the nozzle outlet and its unit is megapascal. *t_i_* is the initiation interval, and its unit is millisecond.

## 4. Discussion

Shock tubes have been widely used to study the explosion resistance of engineering structures and the dynamic mechanical properties of materials. To produce a shock wave that can simulate a free-field explosion, scientists have analyzed the effect of component shape and size on the waveform. Ousji et al. found that a minimum length-to-diameter ratio of 4.5 for the shock tube was required to ensure the planarity of the shock wave [13]. However, the total length of the driver section should not be too long, as a pressure plateau is likely to occur in the blast wave profile [14]. The conical shock tube is a new type of shock tube. It can use smaller charges to obtain stronger shock waves [10] and replicate the deflection patterns of an impulsive blast loading [4]. In addition, the driving method also affects the waveform of the shock wave. Compared to the compression-driven method, the shock wave produced by the explosive-driven method is closer to the waveform of a free-field explosion [3,9]. Ren et al. suggested that the multi-point detonation method reduced the structural strength requirements of the chamber compared to direct detonation in the chamber [18]. However, there is a lack of further experimental studies. In this paper, the shock wave produced by multi-point detonation can better simulate the triangular waveform of a free-field explosion. Different overpressure peaks can be obtained by controlling the detonation interval time. This provides experimental support for the application of multi-point detonation technology in the shock tube.

On the other hand, multi-point detonation has been used more in other areas. Lin et al. conducted single-point detonation tests and four-point simultaneous detonation tests for underwater explosions [20]. At a certain distance, the shock waves from four-point simultaneous detonations are superimposed in the center. This creates a stronger synthetic shock wave. Compared with the aggregate charge, the greater the number of explosion points, the higher the gain of the bubble peak pressure, and the smaller the array distance are, the higher the gain [22]. In multi-source fuel–air explosive cloud explosions, the shock waves converge to form a central high-pressure zone at the field center [23]. In contrast to the above study, in this paper, the multi-point detonation is performed inside the shock tube. Shock waves converge at the axis of the explosion chamber to create a high-pressure zone. In addition, the shock waves are confined by the wall as they propagate. This causes the shock waves to also concentrate on the wall, creating high-pressure areas. This study provides a reference for multi-point detonation studies in confined spaces.

## 5. Conclusions

This paper studied the overpressure field of the shock tube under multi-point simultaneous detonation and multi-point delayed detonation with experiments and numerical simulations. When using a single-point initiation, the load on the wall is evenly distributed, and overpressure at the outlet is the highest. Multi-point simultaneous detonation reduces the overpressure in most areas of the wall, but the maximum overpressure in the focused area of the shock waves is close to that of a single-point detonation. This also reduces the overpressure at the nozzle outlet. Multi-point delayed detonation means that the shock waves generated by each sub explosive chamber cannot all be superimposed, further reducing the overpressure at the wall and nozzle outlet. Comparing the results of the cases, six-point detonation with an interval of 2.5 ms is the best. Compared to single-point detonation, the maximum overpressure on the wall of the explosion chamber was reduced by 62.8%. The peak overpressure at the nozzle exit is 0.62 times that of a single-point detonation.

## Figures and Tables

**Table 1:** Experiments and numerical simulations.

Experiments	Initiation Points	Interval	Simulations	Initiation Points	Interval
1	1	0 ms	1	1	0 ms
2	3	0 ms	2	2	0 ms
3	6	0 ms	3	4	0 ms
4	6	5 ms	4	5	0 ms
5	6	10 ms	5	6	2.5 ms
6	6	30 ms	6	6	7.5 ms

**Table 2:** TNT properties.

Parameters	Density/(kg·m^−3^)	*A*/GPa	*B*/GPa	*R*_1_	*R*_2_	*ω*	*e*/(J·m^−3^)
Values	1630	371.21	3.23	4.15	0.95	0.3	7 × 10^9^

**Table 3:** Air properties.

Parameters	Density/(kg·m^−3^)	*γ*	*e*/(J·m^−3^)
Values	1.225	1.4	2.068 × 10^5^

**Table 4:** Overpressure peak of the experiments and simulations.

Cases	Gauge	Overpressure (MPa)
Experiment 1	1	0.206
Simulation 1	1	0.198
Experiment 1	2	0.159
Simulation 1	2	0.152

## Data Availability

Not applicable.
